# Virucidal Activity of Lemon Juice Against Feline Calicivirus, Surrogate of Norovirus

**DOI:** 10.3390/antibiotics14030273

**Published:** 2025-03-07

**Authors:** Gianvito Lanave, Francesco Pellegrini, Cristiana Catella, Helena Mateos, Gerardo Palazzo, Arturo Gentile, Georgia Diakoudi, Matteo Burgio, Maria Tempesta, Vito Martella, Michele Camero

**Affiliations:** 1 Department of Veterinary Medicine, University Aldo Moro of Bari, 70010 Valenzano, Italy; gianvito.lanave@uniba.it (G.L.); cristiana.catella@uniba.it (C.C.); arturo.gentile@uniba.it (A.G.); georgia.diakoudi@uniba.it (G.D.); matteo.burgio@uniba.it (M.B.); maria.tempesta@uniba.it (M.T.); vito.martella@uniba.it (V.M.); michele.camero@uniba.it (M.C.); 2 Department of Chemistry and CSGI (Centre for Colloid and Surface Science), University Aldo Moro of Bari, 70121 Bari, Italy; helena.mateos@uniba.it (H.M.); gerardo.palazzo@uniba.it (G.P.); 3 Department of Pharmacology and Toxicology, University of Veterinary Medicine, 1078 Budapest, Hungary

**Keywords:** virucidal activity, lemon juice, norovirus, citric acid, disinfection

## Abstract

Noroviruses are a major cause of acute gastroenteritis, often transmitted through contaminated food and water. In this study, lemon juice (LJ), rich in citric acid (CA) and flavonoids, was tested against Feline Calicivirus (FCV), used as a surrogate of human norovirus. Significant virucidal activity was observed for pure LJ (pH = 2.3), with a reduction in viral titers as high as 4.50 log_10_ TCID_50_/50 µL after 30 s and complete inactivation after 1 min. LJ also showed limited virucidal activity at a dilution of 1:2000 (pH = 6.7), with a reduction in viral titer of 0.75 log_10_ TCID_50_/50 µL. CA (at the same molarity as CA in pure LJ and adjusted to pH = 2.3) exhibited virucidal effects comparable to pure LJ, with a decrease in viral titers as high as 3.75 log_10_ TCID_50_/50 µL, whilst diluted CA (pH = 6.7) did not show significant effects. This study demonstrated the virucidal efficacy of LJ, suggesting the role of pH and, eventually, of LJ bioactive compounds against a norovirus surrogate. Due to its large use in food preparation, LJ has the potential to enhance the safety of raw food. Also, broader applications in personal hygiene and surface disinfection could be devised.

## 1. Introduction

Noroviruses (NoVs) are single-stranded RNA viruses that belong to the *Caliciviridae* family. First discovered in 1972, NoVs are common agents associated with acute gastroenteritis in all age groups, and contaminated food and water are a major transmission route [1]. NoV infections occur mainly in community settings, in hospitals, nursing homes, schools, or, typically, in confined environments, such as commercial and cruise ships [2].

NoV cannot be cultivated, which long represented a limitation for diagnostic purposes [3] before the development of rapid diagnostic tests for the detection of NoV nucleic acids or antigens. Yet, the marked genetic/antigenic diversity of NoVs is a challenge for diagnostics. NoVs are classified in genogroups and genotypes. GI and GII noroviruses are the genogroups included in routine testing and most often identified in humans [4].

NoVs are non-enveloped viruses that are very resistant in the environment, surviving at temperatures above 60 °C and even in the presence of chlorine, which is normally used to disinfect drinking water. These viruses are shed in the stools of infected people for at least 72 h after recovery, although viral shedding can last for several weeks. As a result, only strict hygiene practices by food handlers and distributors can effectively prevent virus spread [5,6].

NoV particles spread easily from an infected individual, who is capable of releasing billions of viral particles through feces or oral mucosa, contaminating food, water, and surfaces [7,8,9,10].

Several foods have been frequently associated with NoV outbreaks, including fresh salads, frozen raw fruit and vegetables, cured meats, ice [11,12,13], and bivalve mollusks. Shellfish can accumulate NoVs due to their filter-feeding strategy and are often consumed raw or undercooked [14,15]. The consumption of raw oysters is one of the most common sources of NoV infection [16,17].

Although there are interesting developments in the field of vaccines, the primary strategic approach to limiting the contamination of food, water, and the environment remains strict hand and surface hygiene. Therefore, using effective disinfectants for both hands and surfaces, following the guidelines on technical data sheets for concentrations and contact times, is necessary. Bleach is an excellent disinfectant against NoV, but it can only be used on some surfaces and is not suitable for hand disinfection, due to its irritant action. Alcohol-based disinfectants, widely used for hands, are ineffective according to some studies. Many of the antimicrobial properties found in hand-washing products are attributed to surfactants [7].

Non-conventional approaches for NoV inactivation in food and disinfection are being explored. A promising area of research focuses on natural products, such as fruit or plant extracts, as sources of antimicrobial compounds [18,19].

*Citrus limon* (L.) Burm. f. is an evergreen tree, and the fruit, lemon (Le), yellow in color, is edible, with different varieties distinguished by their shapes. However, the chemical composition of lemon juice (LJ) does not change substantially among different varieties (Table 1) [20].

LJ, unlike Lemon Essential Oil (LEO), has received less attention [21], despite being rich in vitamins and flavonoids with strong antioxidant properties [22]. LJ consumption has been shown to improve metabolism, providing benefits for obesity and diabetes [23,24]. In addition, LJ provides soluble fibers that can influence total cholesterol levels, improving the HDL/LDL ratio [25]. Finally, the content of flavonoids such as hesperidin and naringin has shown antitumoral properties, inhibiting the proliferation of neoplastic cells [24,26,27,28].

The antimicrobial properties of Le products have been well documented against bacteria, yeasts, fungi, and viruses. Specifically, these activities are directed against various bacteria, such as *Enterococcus faecalis*, *Bacillus subtilis*, *Salmonella typhimurium*, *Shigella sonnei*, *Staphylococcus capitis*, *Micrococcus luteus*, *Pseudomonas fluorescens*, *Escherichia coli* [20,29,30], yeasts, fungi [31,32], and viruses such as NoV and herpesvirus [33]. The antimicrobial mechanism of action of LJ is mainly attributed to its low pH, since CA is the most abundant component [20], and the presence of flavonoids such as hesperidin and naringin [20]. These compounds have demonstrated antiviral activity against human herpesvirus 1 and 2, human respiratory syncytial virus, poliovirus, sindbis virus, dengue virus, and hepatitis C virus [34]. The acidity of LJ, due to the high presence of citric acid, creates a hostile environment for many pathogens, inhibiting their growth and proliferation [20,35]. Several studies have suggested that the flavonoids present in LJ inhibit various stages of viral replication [36,37,38]. Furthermore, even sodium citrate (the deprotonated form of citric acid, inhibits several human pathogens at low concentrations [39,40]. It has also been reported that sodium citrate affects the morphology of norovirus-like particles [41].

Since LEO, like all other EOs, has low solubility in water and is biological unstable, it is difficult to use in domestic and industrial sectors [38].

To evaluate the antiviral or virucidal action of any active substances against NoV, laboratories use easily cultivable surrogate viruses with close structural and genetic similarities to NoV, such as Feline Calicivirus (FCV) and murine norovirus (MNV) [33]. The FCV surrogate model has been largely used to study NoV properties, since FCV grows at high titers in vitro on different cell lines. FCV is not a zoonotic virus and can be safely handled in BSL-2 facilities. Also, different FCV strains are available in laboratories and biological banks [42]. The aim of this study was to test the efficacy of LJ against FCV, used as surrogate of human NoV, exploring the possibility of using LJ as a natural, sustainable, and low-cost tool against NoV on surfaces and food.

## 2. Results

### 2.1. Cytotoxicity

**Figure 1:**
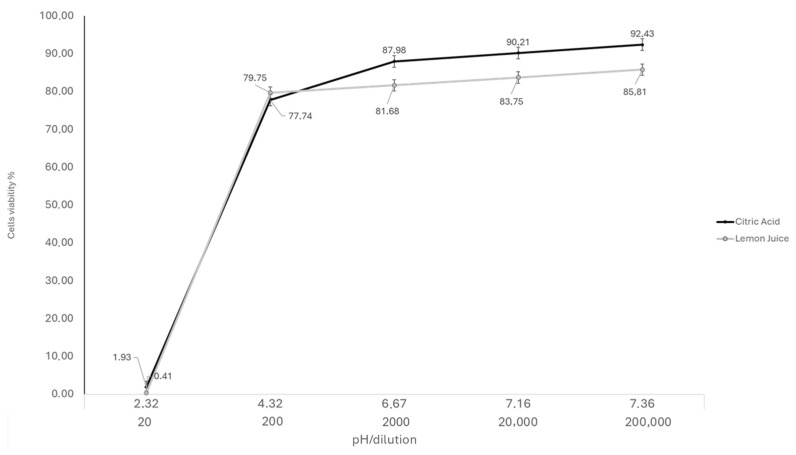
Cytotoxicity of the CrFK cells treated with LJ and CA at 72 h post-treatment and calculated using the XTT assay.

Cytotoxicity was assessed by the measurement of cell viability using the XTT colorimetric method after exposing CrFK cells to various dilutions of LJ (1:20, 1:200, 1:2000, 1:20,000, and 1:200,000) and corresponding CA solutions (pH) for 72 h. Cytotoxicity was assessed by measuring the absorbance signal spectrophotometrically. Based on fitted dose–response curves, the CC_20_ value of LJ was assessed at 1:2000 (pH = 6.7) (Figure 1). When comparing the cytotoxicity on the treated cells of the LJ at concentrations below CC_20_ (1:20, 1:200, 1:2000, 1:20,000 and 1:200,000), the ANOVA model showed a statistically significant decrease in cytotoxicity (F = 211.71, *p* < 0.01). By the pair comparison of individual concentrations, a statistically significant decrease in cytotoxicity was observed between the concentration 1:20 and all other dilutions used (*p* < 0.01). A statistically significant decrease in cytotoxicity was observed between 1:200 and 1:2000, 1:20,000, and 1:200,000 dilutions (*p* < 0.01). Non-statistically significant decreases in cytotoxicity were observed between other concentrations (*p* > 0.05).

### 2.2. Virucidal Activity Assay LJ

**Figure 2:**
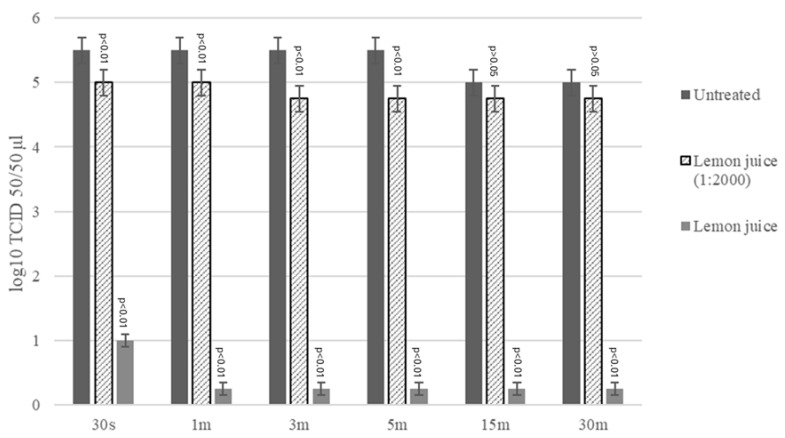
Virucidal effect of LJ incubated with Feline Calicivirus (FCV) for 30 s, 1, 3, 5, 15, and 30 min at room temperature and subsequently titrated in Crandell-Rees Feline Kidney (CrFK) cells. Viral titers of FCV were expressed as log10 TCID_50_/50 μL, and treated virus titers were plotted against the control virus (CV). The bars in the figures indicate the means. The error bars indicate the standard deviation.

The virucidal effects of LJ against FCV were evaluated using the undiluted juice (pH 2.3) and at the maximum non-cytotoxic concentration (1:2000, pH 6.7) at room temperature and at different contact times. The virus treated with undiluted LJ showed a significant reduction in viral titers of 4.50 log_10_ TCID_50_/50 μL after 30 s (*p* < 0.01) and of 5.50 log_10_ TCID_50_/50 μL at 1, 3, 5, 15, and 30 min of contact (*p* < 0.01) (F = 1215.5, DF = 17, CI 95% = 4.517–5.400) (Figure 2).

The virus treated with LJ at the maximum non-cytotoxic concentration (dilution 1:2000), when compared with untreated control virus, showed a significant reduction in viral titers of 0.50 log_10_ TCID_50_/50 μL after 30 s and 1 min (*p* = 0.0204) and of 0.75 log_10_ TCID_50_/50 μL after 3 and 5 min (F = 1215.5, DF = 17, CI 95% = 0.203–0.797) (*p* < 0.01). After 5 min and up to 30 min, the reduction in viral titer was not statistically significant (*p* > 0.05). The virus treated with diluted LJ (dilution 1:2000), when compared with the virus treated with undiluted LJ, showed a significant difference in viral titers of 4.00 log_10_ TCID_50_/50 μL after 30 s, 4.50 log_10_ TCID_50_/50 after 1 min, and 4.25 log_10_ TCID_50_/50 μL from 3 to 30 min (F = 1215.5, DF = 17, CI 95% = 4.132–4.785) (*p* < 0.01) (Figure 2).

### 2.3. Virucidal Activity Assay CA

**Figure 3:**
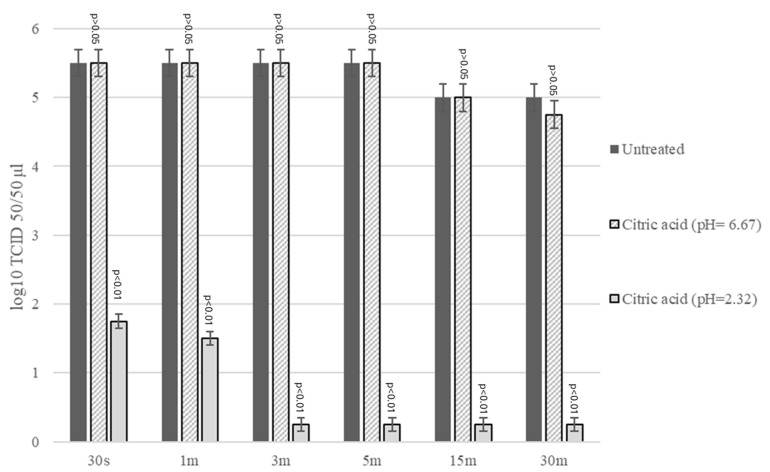
Virucidal effect of citric acid incubated with Feline Calicivirus (FCV) for 30 s, 1, 3, 5, 15, and 30 min at room temperature and subsequently titrated in Crandell-Rees Feline Kidney (CrFK) cells. Viral titers of FCV were expressed as log10 TCID_50_/50 μL, and treated virus titers were plotted against the untreated control virus (CV). The bars in the figures indicate the means. The error bars indicate the standard deviation.

The virus treated with CA at pH 2.3, when compared to the untreated control, showed a reduction of 3.75 log_10_ TCID_50_/50 μL after 30 s (*p* < 0.01), of 4.00 log_10_ TCID_50_/50 μL after 1 min, and of 5.5 log_10_ TCID_50_/50 μL after 3 min of contact (*p* < 0.01) (F = 319.5, DF = 17, CI 95% = 3.791–5.459) (Figure 3). When the virus was treated with CA at pH = 6.7, it showed a non-statistically significant reduction in viral titers of only 0.25 log_10_ TCID_50_/50 μL after 30 min. (*p* > 0.05) (F = 319.5, DF = 17, CI 95% = −0.0939–0.177) (Figure 3). The virus treated with CA at pH 2.3, when compared with the virus treated with solution at pH 6.7, showed a significant difference in viral titers of 3.75 log_10_ TCID_50_/50 μL after 30 s, 4.00 log_10_ TCID_50_/50 after 1 min, 5.25 log_10_ TCID_50_/50 μL after 3 and 5 min, and 4.75 and 4.50 log_10_ TCID_50_/50 μL after 15 min and 30 min, respectively (*p* < 0.01) (F = 319.5, DF = 17, CI 95% = 3.752–5.415) (Figure 3).

## 3. Discussion

Consumers are increasingly oriented toward products of natural origin with proven sustainability and health properties. As a result, industries try to adapt to these trends, developing biological products free of substances perceived as harmful for human health and the environment. For instance, a growing demand for natural disinfectants has risen for various applications, including in the food industry [43].

In this study, LJ was considered, a product that is easily available all year round, inexpensive, organoleptically pleasant, and, above all, natural. Since NoVs are a major agent of acute gastroenteritis [44,45], the effects of LJ were tested against a NoV surrogate, FCV, which can be easily and safely cultured in vitro. We evaluated the virucidal activity of LJ pure and diluted it (1:2000) beyond the maximum non-cytotoxic dose. Pure LJ caused a rapid reduction in virus titer within 30 s, with complete virus inactivation after 1 min. Diluted LJ (at the maximum non-cytotoxic dose) inactivated the virus mildly but significantly after 3 or 5 min, but longer contact times (15 and 30 min) did not decrease further virus infectivity.

LJ is a product rich in CA, 47 g/L [46], with high acidity [47], capable of inactivating various viruses commonly found in food. An acidic pH is a characteristic that can be useful for several applications in the food industry, reducing the need for chemical preservatives. The use of organic acids, particularly citric acid, is widespread in the food industry, and several studies have described their virucidal activity against hepatitis A and E viruses [48,49].

Our study also investigated whether the virucidal activity of LJ is due to CA. To match the conditions of the experiments, we used CA at a concentration (47 g/L, M = 0.24 mol/L) corresponding to the average CA concentration reported for LJ [46]. Also, the pH of CA was adjusted to either 2.3 (the same as pure LJ) or 6.7 (the same as the diluted LJ). A slight delay (after 3 min) in the virucidal activity was observed for CA at pH = 2.3 compared to pure LJ (after 1 min). CA at pH = 6.7 did not show any significant virucidal activity, whilst diluted LJ showed a modest but significant virucidal activity at 30 s, 1 min, 3 min, and 5 min. These pH-independent changes could be due to synergies with other bioactive components of LJ that are not present in CA, such as flavonoids, terpenes, limonene, and hesperidin, known for their antimicrobial and antiviral properties [50].

The virucidal experiments in this study were carried out using 10^6.5^ TCID_50_/50 μL of FCV, corresponding to 3.17 × 10^6^ infectious viral particles per mL. This virus quantity is higher than the NoV loads reported in tissues of naturally contaminated shellfish, which can range between 10^2^–10^4^ genome copies/g of digestive tissues, reaching levels as high as 10^9^ only occasionally [51]. Using human intestinal enteroid cultures inoculated with the fecal samples of patients infected with the GII.Pe-GII.4 Sydney 2012 NoV variant, in qRT-PCR, a cycle threshold cutoff of 30 correlated with infectious NoV [52], indicating that such low NoV contamination is still sufficient to sustain NoV infectivity. Therefore, it could be hypothesized that in most cases of NoV-related food contamination, where NoV titer is low, LJ could be useful to decrease the risks of NoV infection in consumers.

The use of the FCV system as a surrogate of human NoV has some limitations [53]. FCV is thought to have stability in conditions of low pH and heat that is different from that of NoV [42]. Also, a laboratory-adapted strain of FCV was used in the present study. Marked differences in terms of resistance to pH, bile acids, and trypsin have been observed between FCV isolates [54]. Likewise, the virucidal activity of ethanol 70% on NoV infectivity assessed in enteroid cells is strain-specific [55]. Therefore, experiments on the residual infectivity of enteric viruses must be interpreted with some caution.

In several countries, it is common to eat raw seafood. Food contamination can occur at all stages of the supply chain, from primary production to consumption, passing through the processing, transformation, transport, and trade phases [56,57,58]. A common practice among consumers is to eat shellfish products by dispensing a few drops of freshly squeezed lemon. The main reason behind these habits resides in culinary and cultural beliefs, to enhance the taste of the food products, since the acidity of lemon offers an excellent counterbalance to the “salty” taste of seafood, creating a suitable combination.

Despite the unequivocal nutritional value of the non-thermally treated food, the consumption of raw seafood can pose significant health risks to consumers, exposing people to infectious agents other than NoV, such as Astrovirus, hepatitis A Virus, hepatitis E Virus, *Listeria monocytogenes*, *Campylobacter*, *Salmonella*, *Vibrio*, and *Escherichia coli* [59,60,61,62,63,64].

In contexts where the use of chemical disinfectants is limited or not recommended, LJ could also offer a complementary integrative approach for the decontamination of surfaces from non-enveloped viruses such as FCV, and thus for human noroviruses [65].

The virucidal properties present even at non-cytotoxic concentrations open the possibility of integrating LJ extracts into oral hygiene products, such as mouthwashes, or in cosmetic formulations. Furthermore, LJ does not have the disadvantages of LEO, which, as an oil, has a low solubility in water. This particular feature makes it difficult to handle EOs for common domestic and laboratory uses, requiring apolar solvents to make the substance bioavailable to the culture media. When comparing stability, EOs are volatile and sensitive to environmental conditions, with LJ being more resistant and durable [38,66].

## 4. Materials and Methods

### 4.1. Lemon Juice (LJ)

LJ was extracted by squeezing the pulp of lemons of the four seasons variety harvested in June 2024.

### 4.2. Citric Acid (CA)

To evaluate the specific effect of CA, matching solutions with pH levels equal to the undiluted LJ (pH 2.3) and diluted LJ (pH 6.7) were used in our study. To disentangle the effect of pH from the possible effect of the citrate moiety [41], all the solutions had the same molar concentration (M = 0.24 mol/L), which corresponds to the average CA concentration (47 g/L) reported for LJ [46]. The solution at pH = 2.3 was prepared, dissolving the corresponding amount of citric acid and adjusting the pH with NaOH solution. The solution at pH = 6.7 was prepared by mixing a CA 0.24 M solution with a suitable amount of sodium citrate tribasic at the same concentration.

### 4.3. Cells and Viruses

Crandell-Rees Feline Kidney (CrFK) cells cultivated in an incubator at 37 °C in the presence of 5% CO_2_ were maintained in Dulbecco-MEM (D-MEM) (Corning^®^, Glendale, AZ, USA), supplemented with 10% fetal bovine serum, 100 IU/mL penicillin, 0.1 mg/mL streptomycin, and 2 mM l-glutamine. The same medium was used for antiviral tests. The field strain FCV 283/12 was grown and titrated on CrFK cells. The virus stock with a titer of 10^6.50^ Tissue Culture Infectious Dose (TCID_50_)/50 μL was stored at −80 °C and used for all experiments.

### 4.4. Cytotoxicity Assay

The cytotoxicity of LJ and CA were evaluated using the in vitro Toxicology Assay Kit (Sigma–Aldrich Srl, Milan, Italy), based on 3-(4,5-dimethylthiazol-2 yl)-2,5-diphenyl tetrazolium bromide (XTT). The assay was carried out as formerly reported [67]. Confluent 24 h monolayers of CrFK cells grown on 96-well plates were used to assess the cytotoxicity of LJ at different concentrations (1:20, 1:200, 1:2000, 1:20,000, and 1:200,000). The pH of the CA solutions was measured to match the LJ dilutions. The percentage of cytotoxicity was calculated using the following formula: % cytotoxicity = (OD of control cells − OD of treated cells) × 100/OD of control cells. The maximum non-cytotoxic concentration was assessed and regarded as the concentration at which the viability of the treated CrFK cells decreased by 20% with respect to the control cells (CC_20_) [33,68]. The experiments were performed in triplicate.

### 4.5. Virucidal Activity Assay LJ

The possible virucidal effect of LJ against FCV was evaluated by contact with the virus with pure juice (pH 2.3) and a diluted 1:2000 (pH 6.7) solution. One hundred µL of stock virus solution was dissolved in 900 µL of pure and diluted juice at room temperature. After 30 s, 1, 3, 5, 15, and 30 min, the juice/virus and D-MEM/virus (control virus) mixtures were collected and subjected to viral titration on CrFK [68,69]. The experiments were performed in triplicate.

### 4.6. Virucidal Activity Assay CA

Nine hundred microliters of the CA solution at pH 2.3 and 6.7 was combined with 100 μL of stock virus solution. After 30 s, 1, 3, 5, 15, and 30 min, the citric acid/virus and D-MEM/virus (control virus) mixtures were collected and subjected to viral titration on CrFK [68]. The experiments were performed in triplicate.

### 4.7. Viral Titration

Ten-fold dilutions (up to 10^−8^) of each supernatant were titrated in quadruplicates in 96-well plates containing CrFK cells. The plates were incubated for 72 h at 37 °C in a 5% CO_2_ environment. Based on the cytopathic effect, the viral titer was calculated [69,70]. The experiments were performed in triplicate.

### 4.8. Data Analysis

LJ concentrations were converted into log_10_, and cytotoxicity assays results were evaluated using a non-linear curve fitting. Moreover, a dose–response curve was elaborated through a non-linear regression analysis to evaluate goodness of fit. From the fitted dose response curves achieved in each experiment, CC_20_ was assessed. The normality of distribution was evaluated by Shapiro–Wilk test (*p* > 0.05). Data from cytotoxicity and virucidal activity assay were expressed as the mean ± SD and analyzed using a One-way Analysis of Variance (ANOVA) followed by a Bonferroni test as a post hoc test (statistical significance set at 0.05). Statistical analyses were carried out using the GraphPad Prism v10.2.1 program (Dotmatics, Boston, MA, USA).

## 5. Conclusions

Integrating LJ into existing disinfection protocols will require a precise evaluation of the optimal protocols, assessing, for instance, the contact time, concentration, and formulations required to enhance LJ activity. Also, using LJ in the processing/preparation of raw food could benefit from LJ antimicrobial properties, taking advantage of its sensorial and organoleptic qualities.

## Figures and Tables

**Table 1:** Composition of lemon juice.

Group of Compound	Metabolites
Flavonoids	flavonones: hesperidin, naringin flavones: apigenin, chrysoeriol, diosmetin, luteolin flavonols: isoramnethin, quercetin, rutoside dihydroxyflavonols: dihydroxyisoramnethin-7-O-rutinoside
Phenolic acids	Ferulic acid, synapic acid
Vitamins	vitamins: C (53 mg/L), A, B_1_, B_2_, B_3_

## Data Availability

All the data produced and mentioned in this paper are freely available to the scientific community.
